# Effect of Abiotic Treatments on Agricultural Plastic Waste: Efficiency of the Degradation Processes

**DOI:** 10.3390/polym16030359

**Published:** 2024-01-29

**Authors:** Zbigniew Emil Blesa Marco, José Antonio Sáez, Francisco Javier Andreu-Rodríguez, Rosa Penalver, Manuel Rodríguez, Kristina Eissenberger, Patrizia Cinelli, María Ángeles Bustamante, Raúl Moral

**Affiliations:** 1 Centro de Investigación e Innovación Agroalimentaria y Agroambiental (CIAGRO-UMH), Universidad Miguel Hernández, EPSO, Ctra. Beniel Km 3.2, E-03312 Alicante, Spain; zblesa@umh.es (Z.E.B.M.); j.saezt@umh.es (J.A.S.); raul.moral@umh.es (R.M.); 2 Department of Analytical Chemistry, Faculty of Chemistry, Regional Campus of International Excellence “Campus Mare Nostrum”, University of Murcia, E-30100 Murcia, Spain; 3 Department of Ingeniería Química, University of Alicante, P.O. Box 99, E-03080 Alicante, Spain; manuel.rodriguez@ua.es; 4 Sustainable Packaging Institute SPI, Faculty of Life Sciences, Albstadt-Sigmaringen University, Anton-Günther-Str. 51, 72488 Sigmaringen, Germany; eissenberger@hs-albsig.de; 5 Department of Civil and Industrial Engineering, University of Pisa, 56126 Pisa, Italy; patrizia.cinelli@unipi.it

**Keywords:** agri-food waste plastic, polystyrene, polyethylene terephthalate, linear low density of polyethylene, low density polyethylene, thermal analyses, FTIR, e-beam

## Abstract

In this study, four different plastic materials usually used in the agricultural sector (polystyrene film (PS), polyethylene terephthalate film (PET), low-density polyethylene film (LDPE) and linear low-density polyethylene film (LLDPE)) were subjected to different abiotic treatments, including photo-oxidation (ultraviolet and e-beam radiation) and thermochemical treatments, to enhance polymer degradation. The extensive use of these polymers leads to large amounts of plastic waste generation, including small plastic pieces, known as microplastics, which affect the quality of the agricultural environment, including soil fertility and quality. Therefore, polymer degradation strategies are needed to effectively reduce plastic waste to protect the agricultural sector. The degree of polymer degradation was assessed by the use of thermal and spectroscopic analyses, such as TGA and FTIR. In addition, efficiency, cost–benefits, and potential side-effects were also evaluated to propose the optimal degradation strategy to reduce plastic waste from the point of view of efficiency. The results obtained showed that the pre-treatments based on photo-oxidation (ultraviolet B and C and e-beam radiation) were more efficient and had a better cost–benefit for the degradation of the polymers studied in relation to the thermochemical treatments. Specifically, ultraviolet photo-oxidation worked well for PS and PET, requiring low energy and medium times. However, e-beam radiation was recommended for PE (LDPE and LLDPE) degradation, since high energy and long times were needed when ultraviolet energy was applied to this polymer. Furthermore, the overall efficiency of the plastic degradation of pre-treatments should be studied using a multicriteria approach, since FTIR assessments, in some cases, only consider oxidation processes on the plastic surface and do not show the potential integrity changes on the plastic probes.

## 1. Introduction

Since 1950, plastic production has increased at an average rate of almost 10% every year on a global basis [1]. The great diffusion of plastic in our society is due to a range of characteristics of plastic materials, such as their low density, lightness, strength, workability, and low cost compared to other materials. However, such diverse consumption leads to a diverse waste stream [2]. Plastic materials are widely used in European agriculture because they contribute to increasing the quality and the quantity of production. About twenty distinct groups of plastics for agricultural use exist, each with various formulations available to enable the best choice for each specific application. The main polymers used in agricultural activities are polyolefins such as polyethylene (PE) and polypropylene (PP), due to their low cost, good workability, high impact resistance, excellent chemical resistance and electrical insulation properties. They are mainly used to produce films (for greenhouses, low tunnels, mulching, and silage), due to their high tear and impact strength [3].

Regarding the food packaging market, the European total demand for plastic has risen to 49 million tonnes per year, of which 37–38% is used for packaging. The packaging plastics consumed worldwide account for 35% of the total [4]. Around 60% of all plastic packaging is used for food and beverages, while the rest covers non-food applications, such as healthcare, cosmetics, consumer, household, apparel and shipment packaging; the five most commonly used polymers are PE, PP, polystyrene (PS), terephthalate of polyethylene (PET) and polyvinyl chloride (PVC) [5]. Therefore, of the total plastic waste, 60% corresponds to packaging and 5% corresponds to agricultural plastic waste. The high global volume of plastic waste is difficult to manage, and it has a negative effect on different ecosystems. Therefore, it is clearly necessary to develop innovative approaches to reducing plastic residues, which would support the use of packaging and agricultural plastics in an environmentally friendly way. Plastic remediation strategies are key to protecting the agricultural sector, as well as human health. In this study, polymer degradation was the assessed remediation approach. 

In a polymer, any physical or chemical change in its properties is a result of environmental factors, such as light, heat, moisture, chemical conditions or biological activity. Thus, the processes inducing changes in polymer properties (the deterioration of functionality) resulting in bond scission and subsequent chemical transformations are known as polymer degradation. Considering the complexity of plastic degradation and the long degradation times of plastic waste under natural conditions, this work is focused on the assessment of the most efficient pre-treatment procedure to enhance subsequent biotic plastic degradation. In this study, the applied pre-treatments were abiotic procedures.

Mantia et al. (2017) [6] grouped the main abiotic treatments used to degrade plastics in three main types:

- Thermal/thermomechanical treatments;
- Photo-oxidation treatments;
- Chemical/thermochemical treatments.

**Figure 1:**
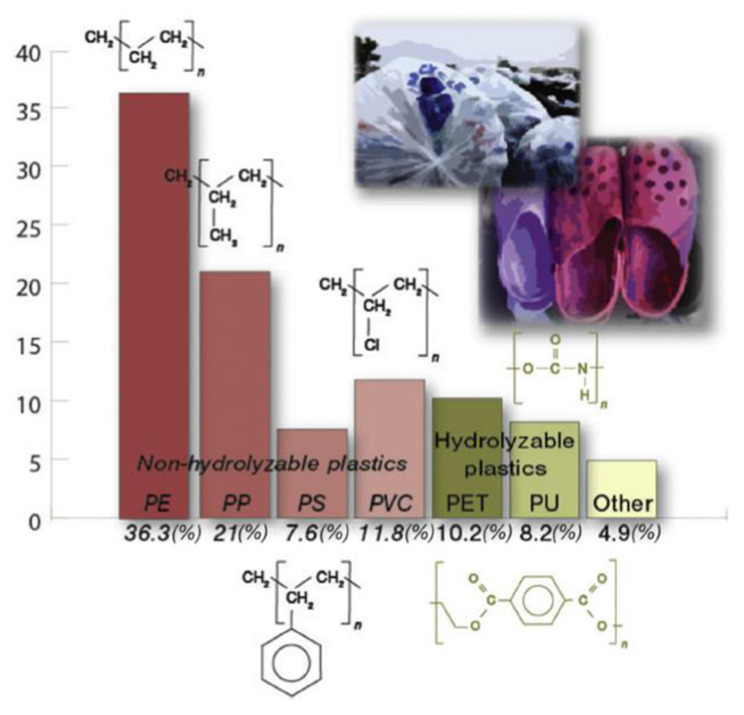
Market shares of the six commercially most important plastic material types [7]. Their monomer structures in high-molecular-weight polymer chains are shown adjacent to bars. Abbreviations: PE, polyethylene; PET, polyethylene terephthalate; PP, polypropylene; PS, polystyrene; PU, polyurethane; PVC, polyvinyl chloride (Reproduced with permission from Hedda Inderthal, Siew Leng Tai, Susan T.L. Harrison, Elsevier, 2021).

Thermal treatments are mainly based in the action of heat; thus, the degrading effects can be very different, depending on the components of the blends and on their chemical structures. Usually, degradation can be facilitated by reducing crystallinity and packaging, enhancing chain mobility and stress. The combination of mechanical stress with thermal variation results in a “thermomechanical” degradation. Photo-oxidation treatments are based on the degrading action of the electromagnetic radiation of polymers. The energy of the radiation, correlated with specific wavelengths, results in the breaking of specific chemical bonds into the polymer structure. Therefore, depending on the energy, different types of chemical bonds can be broken, initiating degradation cascades. Chemical/thermochemical treatments are based on the action of acidic or alkaline conditions, polar or non-polar solvents and specific chemical compounds (initiators) on the integrity of polymers and blends. This kind of treatment can be very efficient in specific polymers, e.g., the use of aromatic amines in PE. The combination of chemicals and heat induces significant increases in degradability. Thus, the process of plastic degradation is determined by both environmental conditions and the physico-chemical properties of polymeric substances. The physico-chemical properties of plastic play an important role in the degradation process, especially its chemical structure and its hydrolysable capacity. Figure 1 shows the market shares of the most significant plastics, including their hydrolysable capacity [7]. 

The degradation of polymers by abiotic and biotic treatments have been studied. During polymer degradation, the polymer carbon backbone is oxidized and fragmented into oxygenated fragments [8]. In the case of PE, although it does not contain heteroatoms nor double bonds in its structure, the photo-degradation process may be initiated by the presence of small amounts of external impurities [9]. The photo-oxidation of PE and PS waste was studied by using fluorescent UV lamps at different temperatures and times [10,11].

On the other hand, there are few studies related to the thermochemical degradation of plastic waste. The thermochemical degradation of PE and PS with different catalysts has been reported [12]. However, the thermochemical degradation of other polymers, such as PET, included in this study have scarcely been evaluated. 

Regarding biotic strategies, a wide bacterial biodiversity has been reported to degrade plastics. The maximum biodegradation of several synthetic plastics for microbial pure cultures has been summarized in the literature [13]. A microbial consortium demonstrated its capacity to enhance the degradation of several polymers, such as PE and LDPE, with weight reductions of 81% after 120 days [14]. On the other hand, oxygen-containing polymers, such as PET, have been demonstrated to be more susceptible to biodegradation [15]. The main limitations on the biological degradation of plastics are due to their high molecular weight, lack of favorable functional groups, and high degree of crystallization. Therefore, a previous abiotic treatment, such as hydrolysis and/or oxidation, would overcome these limitations, making the bulk of the polymer more available for biological attack [16,17].

Thus, techniques such as scanning-electron microscopy (SEM), Fourier transform infra-red spectroscopy attenuated total reflection (FTIR-ATR), hydrogen nuclear magnetic resonance (H-NMR), differential calorimetry scanning (DSC) and thermogravimetry coupled to mass spectrometry (TGA-MS) have been commonly applied to provide structural information during polymer degradation [18]. Other techniques, such as weight loss, C, H and N analyses or respirometer studies (CO_2_ released during the degradation process) are the most common analytical methodologies applied to assess the degradation level of PBAT-PLA due to their simplicity and low cost [19]. 

Unlike most previous studies, which focused on one polymer, in this work, two groups of polymers from different chemical families usually used in the agricultural sector and classified according to their resistance to degradation were considered: C-C backbone polymers, such as PS and PE (low-density PE (LDPE) and linear low-density PE (LLDPE)), as well as an oxygen-containing polymer (PET) mainly applied in the packaging market. Selected samples of each type of polymer were subjected to different abiotic treatments, including photo-oxidation with UV light and a more advanced instrumental procedure based on e-beam radiation and thermochemical treatments, to facilitate subsequent polymer biodegradation. The degradation was evaluated using a multicriteria analytical approach based on thermal and spectroscopic analyses, such as TGA and FTIR. It is worth mentioning the importance of the application of different analytical techniques, which is barely used in the literature, to consider not only oxidation processes on plastic surfaces, but also potential integrity changes on plastic probes. In addition, efficiency, cost–benefits and potential side-effects were also evaluated to propose the optimal degradation strategy to reduce plastic waste from the point of view of efficiency. 

## 2. Materials and Methods

### 2.1. Polymers Used

Four different pure plastic materials were considered for this study, most of which are used in agricultural activities: polystyrene film (PS), polyethylene terephthalate film (PET), low-density polyethylene film (LDPE) and linear low-density polyethylene film (LLDPE), all in the virgin form. All these materials were provided by the University of Pisa (UNIPI) in the framework of the research project RECOVER, financed by the BBI-JU-Horizon 2020. 

### 2.2. Processing Conditions to Enhance Polymer Degradation

#### 2.2.1. Photo-Oxidation: UV Pre-Treatments

The photo-oxidation treatments considered were based on the exposure of plastic films to different UV ranges and duration. Thus, the plastic probe samples (4–20 cm^2^) of PS and PET were subjected to 120 h of exposition, while LDPE and LLDPE were exposed during 750 h, under UV radiation at different wavelengths (UV-B at 320 nm and UV-C at 253 nm). The chambers used were designed by the GIAAMA research group at Miguel Hernández University (Orihuela, Spain) and had the following characteristics: (a) UV chamber B used 2 Philips TL 20 W/01 RS SLV/25 320 nm lights, with a surface (S) of 0.67 × 0.37 = 0.2479 m^2^, which worked with a power of 161.35 W/m^2^; (b) UV chamber C used 2 Philips TUV T8 F17 1SL/25 253 nm lights, with S = 0.67 × 0.37= 0.2479 m^2^, and worked with a power of 137.15 W/m^2^. The details of the UV treatments (B and C) are shown in Table 1. 

#### 2.2.2. E-Beam-Radiation Pre-Treatment

For the application of the e-beam radiation to the plastic materials, we used an EBlab 200 device (Comet, Wünnewil-Flamatt, Switzerland), specialized for low-energy electron-beam treatment; the device harbors an EBA-200 e-beam lamp operating at accelerating voltage from 70 to 200 KeV, which generates a curtain-like electron beam about 23 cm in width, corresponding to a total dose energy from 100 to 1000 kGy, and allows the irradiation of specimens 0.21 m × 0.3 m in size in one run, which takes a maximum of 20 s. The analyses were carried out in ambient atmosphere. The plastic samples used were plastic probes with maximum dimensions of 21 cm × 30 cm. 

#### 2.2.3. Thermochemical Pre-Treatments

The thermochemical pre-treatments used to favor plastic degradation were based on the exposition of plastic films to several oxidizing agents, including different conditions of reaction temperature, attack duration and oxidizer concentration. Table 2 summarizes the different reagents and the conditions used for the chemical degradation of the polymer samples.

### 2.3. Analytical Approaches to Evaluating Polymer Degradation 

Different approaches were considered to stablish the potential degree of the damage of the pre-treatments to the selected plastic samples.

#### 2.3.1. FTIR Determinations

To monitor the evolution in the chemical structures of the polymer samples due to the formation or disappearance of specific functional groups, infrared analyses of the samples in x mode were carried out. An IFS 66/S spectrometer (Bruker Corporation, Ettlingen, Germany) with a resolution of 1 cm^−1^ was used. It had a source, a KBr beam splitter and two detectors: a DLaTGS detector at room temperature for routine measurements and to obtaining spectra between 7000 and 400 cm^−1^ and another MCT detector cooled with liquid nitrogen with high sensitivity and speed of measurement, ideal for action kinetics between 600 and 4000 cm^−1^. In addition, a FTIR 4700 spectrometer (JASCO International Co., Ltd., Tokyo, Japan) with a resolution of 0.5 cm^−1^, was also used. It had a source of go media, a KBr beam splitter encapsulated in Germanium and a DLaTGS detector for routine measurements between 7800 and 400 cm^−1^. The FTIR spectra have been widely used to evaluate plastic degradation by determining several indexes [20,21,22]. These indexes are defined as the ratio between the integrated band absorbance of the functional group (or specific bond type), which may result from polymer degradation, and that of a reference peak used to characterize the degree of oxidation of the considered polymer (Equation (1)):

Functional group index = (Absorbance functional group peak)/(Absorbance reference peak),(1)

According to the values obtained in these indexes, the higher the values, the greater the degree of degradation of the polymer. Thus, the carbonyl index, defined by [11] as the absorbance of the carbonyl group at 1720 cm^−1^ versus the absorbance of the reference peak at 2920 cm^−1^, which corresponds to alkane C-H stretching vibrations was the main index used in this study to evaluate polymer degradation, since the presence of carbonyl groups is an indicator of polymer degradation [23,24]. Furthermore, additional indexes (the lactone index, carboxyl index, ceto carbonyl index, ester carbonyl index and hydroxyl index) [20,21,25,26] were also considered in the statistical study.

#### 2.3.2. Thermal Analysis

Thermogravimetric analysis (TGA) was performed using a Q500 thermogravimetric analyser produced by TA Instruments (New Castle, DE, USA), under nitrogen atmosphere, on about 2 mg of sample, from room temperature to 700 °C, with a heating rate of 10.00 °C/min.

### 2.4. Statistical Analysis

The analysis of the data of the main indexes was carried out using Pareto charts, bar charts ordered by frequency counts, from highest to lowest. Pareto diagrams reflect the ordered frequency counts of values at different levels of a categorical or nominal variable, based on the 80/20 rule, which considers that 80% of the effects are derived from 20% of the causes. The Pareto diagrams were obtained using the IBM SPSS Statistics v. 27.0 software (IBM SPSS Inc., Chicago, IL, USA).

## 3. Results and Discussion

The main results obtained after the application of the different pre-treatments are focused on the effects on each type of plastic, considering the corresponding FTIR indexes that best explained the changes observed, and the efficiency, cost–benefits, and potential side-effects were also evaluated to propose the optimal degradation strategy.

### 3.1. Effects of the Different Pre-Treatments on PS Materials 

#### 3.1.1. Effects of the Photo-Oxidation Pre-Treatments

**Figure 2:**
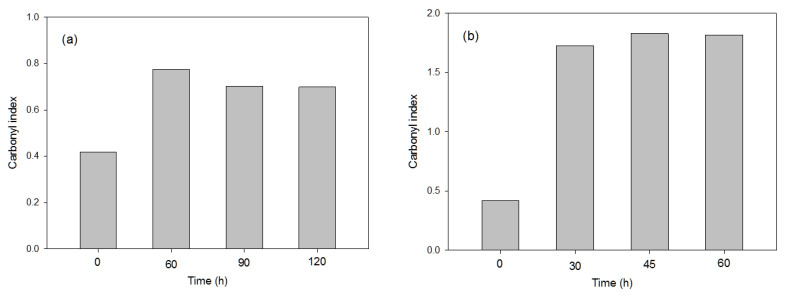
Values of FTIR carbonyl index used in the representative PS sample after applying, for different exposure times, the following UV pre-treatments: (**a**) UV-B; (**b**) UV-C.

The photo-oxidation pre-treatments studied for polymer degradation were UV-B, UV-C and e-beam radiation, while considering, in the latter, four different radiation levels (total dose), from 100 to 500 kGy. As previously noted, FTIR indexes reflect the degree of degradation of a polymer, in this case, e.g., the higher the carbonyl index, the greater the degree of polymer degradation, since more carbonyl groups are generated. Figure 2 shows the carbonyl index obtained from the PS polymer under UV-system types B and C under different times. 

The pre-treatment UV-C produced the highest polymer degradation with an exposure time of 45 h, reflecting that this pre-treatment achieved the highest PS degradation compared to the application of UV-B (at any exposure time) and of e-beam radiation, which only showed a slight increase in the carbonyl index when the total dose increased, with the carbonyl index values lower than 0.4 in all cases (Table 3). This could have been due to the lower wavelength (low UV region) provided for the type C spectrophotometer (253 nm) compared with the type B (320 nm). These results agree with those observed in previous works, since the presence of unsaturated double bonds in PS makes this polymer more susceptible to photo-degradation [27,28].

To validate the FTIR results, TGA analyses of the PS virgin and degraded samples after the application of the photo-oxidation treatments were carried out, and the main results are shown in Table S1. The same conclusions were obtained by comparing the degradation-onset temperature in the TGAs of the different samples. Specifically, a decrease in the onset temperature of 26.6 °C was observed when the UV-C beam was used for 30 h versus the virgin PS; this degradation pre-treatment was the most efficient. In the case of the e-beam pre-treatment, although the carbonyl index values did not show degradation evidence, the TGA results demonstrated (Table S1) that the e-beam radiation enhanced the PS degradation, with a decrease in the onset temperature of almost 20 °C (residue increase of 1.5 wt.%) for an e-beam radiation of 600 kGy. Thus, although it is known that FTIR-related techniques are very useful for detecting surface changes, significant modifications to the entire plastic sample are better shown by TGA data. Therefore, it is important to combine different analytical techniques to obtain a complete evaluation of the polymer-degradation processes.

#### 3.1.2. Effects of the Thermochemical Pre-Treatments

**Figure 3:**
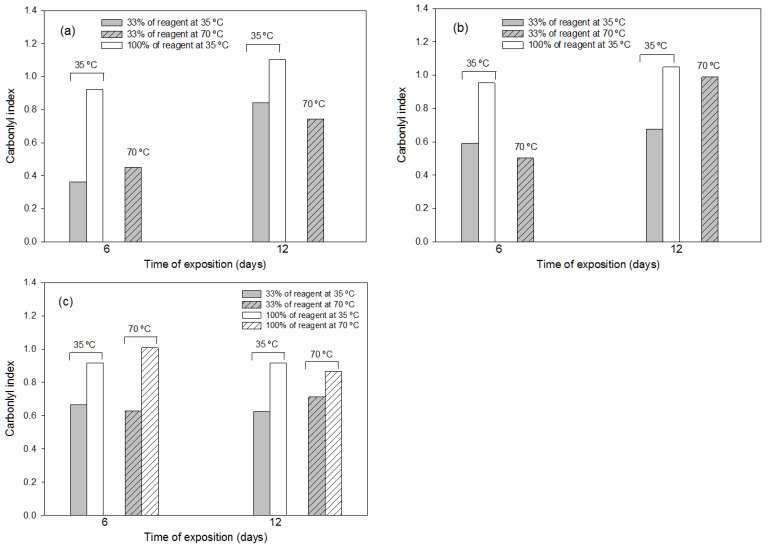
Values of FTIR carbonyl index used in the representative PS sample after application for different exposure times (6 and 12 days), at different temperatures (35 °C and 70 °C) and at different reagent concentrations of the following thermochemical treatments: (**a**) treatment with ammonium persulfate; (**b**) treatment with chromic mixture; (**c**) treatment with aqua regia. In the treatment without bar, at least 50% (dry-matter basis) of plastic sample disintegrated.

The influence of the different thermochemical conditions applied to the representative PS samples on the carbonyl index values are shown in Figure 3a–c. The pre-treatments with ammonium persulfate and chromic mixture showed the strongest oxidizing effect, since these reagents at 100% concentrations seemed to produce the highest amount of degradation in the PS sample, with a disintegration of at least 50% of the plastic sample observed at the exposure temperature of 70 °C, independently of the exposure time (6 or 12 days). Therefore, heating is not needed if pure reagents (ammonium persulfate or chromic mixture) are used for PS degradation over 6 days at 35 °C. Thus, in general, the temperature condition of 35 °C produced more efficient degradation when the reactive was pure (100%), showing, in all cases, a high degradation effect. Longer degradation times (12 days) were also assessed, and similar results were obtained, which means that times no longer than one week are needed to obtain efficient PS degradation. 

Pareto charts (Figure S1) for other FTIR indexes (lactone index, carboxyl index, ceto carbonyl index, ester carbonyl index and hydroxyl index) with the aqua regia pre-treatment (for the rest of chemicals, it was not possible to carry out statistical analyses due to polymer disintegration under certain conditions) were produced to evaluate the factor (time–A, temperature–B or reagent concentration–C) with the highest effect on the polymer degradation. The statistical analysis shows that the concentration of the chemical reagent was the most significant factor t affect the PS degradation, followed by the interaction of the three factors (temperature, time and concentration).

### 3.2. Effects of the Different Pre-Treatments on Polyolefin (LDPE and LLDPE) Materials 

Polyolefins, such as linear low-density polyethylene (LLDPE) and low-density polyethylene (LDPE), are used in many different markets due to their special properties, low production costs, light weights, and high chemical resistance. However, polyolefins are very resistant plastics, with high biological and chemical inertness, whose biodegradation and natural degradation occur at very slow rates and only to a limited extent [29].

#### 3.2.1. Effects of the Photo-Oxidation Pre-Treatments

**Figure 4:**
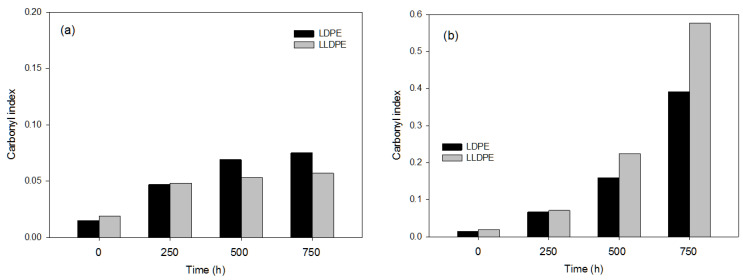
Values of FTIR carbonyl index used in the representative LDPE and LLDPE samples after applying, for different exposure times, the following UV pre-treatments: (**a**) UV-B; (**b**) UV-C.

Due to the higher resistance of the polymers LDPE and LLDPE, the exposure time considered for the UV pre-treatments and the energy applied with the pre-treatment via the e-beam were higher than in the plastic types previously studied. The most notable effect on the LDPE and LLDPE materials was observed for the UV-C pre-treatment at the highest exposure time (750 h) (Figure 4a,b), which indicates that this pre-treatment produced the highest degradation in both polymers, with very low values of the carbonyl index observed for the e-beam pre-treatment (Table 3). These results indicate the high resistance of these specific polymers to photo-oxidation due to their highly stable chemical structures [30]. The chemical and physical changes occurring during the LDPE and LLDPE degradation process were correlated with the energy needed to initiate the removal of water from the bulk of the polymers. Furthermore, since LDPE and LLDPE do not contain any unsaturated double bonds, these polymers have a higher resistance to photo-degradation, but the presence of small amounts of external impurities or structural abnormalities can allow the initiation of the photo-degradation process [31].

The effect of the e-beam was not reflected in the carbonyl index values for either polymer, but the TGA data showed an increase in the degradation of both the LDPE (Table S2) and the LLDPE (Table S3), with a decrease in the onset temperature of almost 15 °C and a residue increase (1.58 wt.%) in the sample degraded by the e-beam (450 GKy) compared with the virgin LLDPE sample. Although the e-beam treatment worked for both the LDPE (Table S2) and the LLDPE (Table S3) a higher effect on the degradation of the LDPE than the LLDPE was found. This can be explained by the slightly lower crystallinity of the LDPE compared with that of the LLDPE, which means more flexibility in the molecular chains, facilitating degradation rates. Both the UV and the e-beam pre-treatment affected the thermal behavior of the LDPE and LLDPE samples. Moreover, the longer the UV treatment time or the higher the e-beam energy, the lower the LDPE and LLDPE thermal stability. However, the efficiency of the treatments was different depending on the polymer, with e-beam being the more effective treatment for the LDPE, and the UV-C for the LLDPE, as reported by Jeon and Kim (2014) [32] in a study of the degradation of LLDPE exposed to UV irradiation.

#### 3.2.2. Effects of the Thermochemical Pre-Treatments

**Figure 5:**
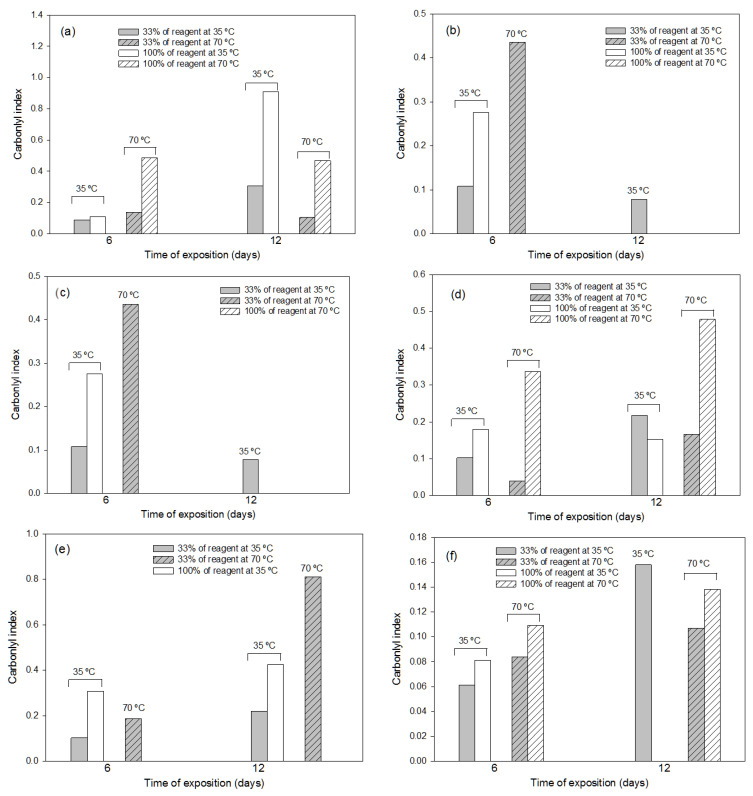
Values of FTIR carbonyl index used in the representative LDPE (**a**–**c**) and LLDPE samples (**d**–**f**) after application, for different exposure times (6 and 12 days), at different temperatures (35 °C and 70 °C) and at different reagent concentrations, of the following thermochemical treatments: (**a**) treatment of LDPE with ammonium persulfate; (**b**) treatment of LDPE with chromic mixture; (**c**) treatment of LDPE with aqua regia; (**d**) treatment of LLDPE with ammonium persulfate; (**e**) treatment of LLDPE with chromic mixture; (**f**) treatment of LLDPE with aqua regia. In the treatments without bar, at least 50% (dry-matter basis) of plastic sample disintegrated.

The thermochemical pre-treatments were more efficient in LDPE degradation than photo-oxidation, but not in LLDPE degradation. Overall, the pre-treatments with the three oxidants at the highest concentration (pure reagent, 100%) produced the greatest degradation for both polymers, but the exposure conditions at which this effect was observed were different. Disintegration in both polymers was only observed for the chromic mixture and aqua regia pre-treatments, at the highest concentration (100%) and temperature (70 °C) at any exposure time (6 or 12 days) for the chromic mixture, and at the highest concentration and lowest temperature (35 °C) over 12 days for the aqua regia (Figure 5a–c for LDPE and Figure 5d–f for LLDPE), showing that the pure chromic mixture was overly aggressive, especially for the LDPE polymer, producing disintegration in most of the tests. Without considering the disintegration effect, in general, the highest degradation of LDPE was found at the lowest concentration of reagent (33%) at exposure condition of 70 °C over 6 days when the chromic mixture or aqua regia were used in the pre-treatment. However, for the LLDPE, the best results were obtained when the chromic mixture was used for 12 days at 70 °C with a concentration of 33%, since the pure reagent produced sample disintegration. The Pareto charts (Figures S2 and S3 for LDPE; Figures S4 and S5 for LLDPE) showed similar results for both polymers, with the exception of the aqua regia reagent, for which the exposure temperature was more significant for polymer degradation than for chemical reagent concentration. 

The Pareto charts corresponding to the FTIR indexes of LDPE obtained after the pre-treatments with ammonium persulfate and aqua regia (the disintegration of several samples with the pre-treatment with the chromic mixture made it impossible to conduct statistical analyses), showed that the concentration of the chemical reagent was the most significant factor for polymer degradation, followed by the combination of time and temperature (Figures S2–S5). 

### 3.3. Effect of the Different Pre-Treatments on PET Materials 

#### 3.3.1. Effects of the Photo-Oxidation Pre-Treatments

**Figure 6:**
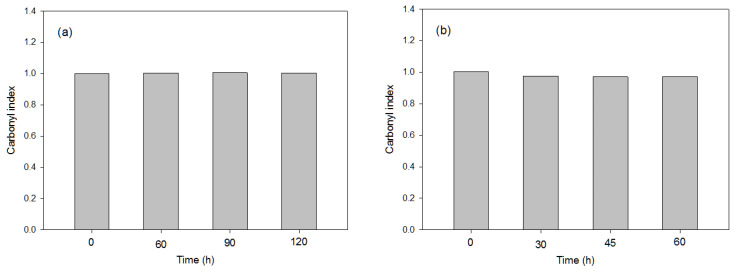
Values of FTIR carbonyl index used in the representative PET sample after application, for different exposure times, of the following photo-oxidation pre-treatments: (**a**) UV-B; (**b**) UV-C.

The greatest carbonyl index (0.84) value was obtained with the pre-treatment using UV-B over an exposure time of 90 h (Figure 6a,b). Therefore, further exposure times under the UV-B beam are not needed; 90 h is the optimal exposure time to achieve maximum polymer degradation. On the other hand, the UV-C beam did not produce any polymer degradation, nor did the e-beam radiation, and these treatments were discarded. The presence of the carbonyl group in the polyester structure of PET facilitates photo-oxidation, indicating that PET degrades rather rapidly when it is exposed to UV light, as reported in other works. Several studies have suggested that the photo-oxidation of PET involves the formation of hydroperoxide species through the oxidation of the CH_2_ groups adjacent to the ester linkages and the hydroperoxide species, involving the formation of photo-products through several pathways [33]. Thus, the oxidation of the -CH_2_ groups, which produces an increase in -C=O groups in the aliphatic chains, is mainly described in the 1470–1740 cm^−1^ absorption band ratio [34]. Regarding the TGA results, a slight decrease in the onset temperature was observed when the UV-B beam was used for 90 h compared with the virgin PET (Table S4). This result supports previous studies that reported the strong effect of UV-B beam on PET-degradation efficiency. On the other hand, the TGA also showed no clear differences with the e-beam pre-treatment (Table S4), which highlighted the low-efficiency degradation using this procedure for PET. 

#### 3.3.2. Effects of the Thermochemical Pre-Treatments

**Figure 7:**
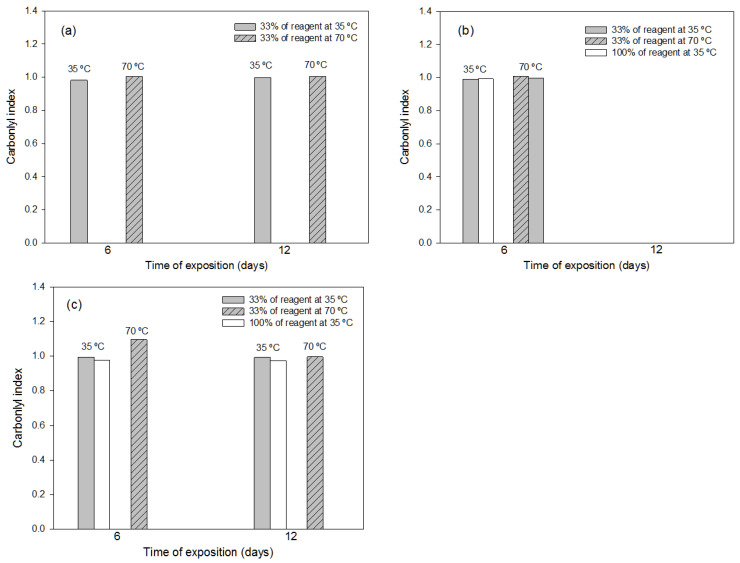
Values of FTIR carbonyl index used in the representative PET sample after application, for different exposure times (6 and 12 days), at different temperatures (35 °C and 70 °C) and at different reagent concentrations, the following thermochemical treatments: (**a**) treatment with ammonium persulfate; (**b**) treatment with chromic mixture; (**c**) treatment with aqua regia. In the treatment without bar, at least 50% (dry-matter basis) of plastic sample disintegrated.

The degradation effect on the PET material was mainly dependent on the chemical reagent used and on the concentration of this reagent (Figure 7a,b). 

In general, the highest concentration (100%) seemed to produce the greatest polymer degradation, even with the disintegration of the plastic, depending on the reagent and on the exposure conditions. If the disintegration effect is not considered, in general, the greatest degradation of PET was observed at the lowest concentration of reagent (33%) at the exposure condition of 70 °C during 6 days with any of the chemical reagents considered. Pure chemical reagents produce the disintegration of the plastic material in most cases; therefore, it is recommended to use the diluted reagent. Thus, any of the three studied chemical reagents at 33 wt.% (ammonium sulphate, chromic mixture and aqua regia) can be used for PET-degradation purposes, using a temperature of 70 °C for 6 days to ensure the degradation of the whole polymer while avoiding disintegration. As all the pure oxidants produced sample disintegration, it was not possible to carry out statistical analyses based on Pareto charts.

### 3.4. Selection of the Most Efficient and Highest-Cost–Benefit Pre-Treatment Procedure

The overall efficiency of the pre-treatments used on plastic degradation must be obtained using a multicriteria approach, because FTIR assessments of pre-treatments, in some cases, only consider oxidation processes on the plastic surface and do not show the potential integrity changes on the plastic probes. In this context, e-beam irradiation seemed to be more efficient when TGA was also used to monitor plastic modifications. To carry out a cost-effective analysis of the different pre-treatments, we considered a qualitative classification based on three levels of costs (Table 4). The cost related to time was calculated depending on the time required to achieve the optimal result by each of the pre-treatment approaches, expressed in days; the cost linked to energy consumption was expressed in energy/m^2^ as a normalized approach according to our assays, considering for the UV treatments the energy consumption of the UV lamps and, for the e-beam, the energy required to apply the e-beam dose; both were normalized by the irradiated surface in the equipment. In the case of the thermochemical pre-treatments, we only considered the energy consumption of the temperature-control and stirring operations. The assessment of environmental costs is complex, but we applied a simplified hierarchical approach, including the binary consideration of the followed items: greenhouse gases potential emissions (yes/no), use of water (yes/no), waste production (yes/no) and energy consumption (yes/no). We scored each yes point as 1 and each no as a 0, with lower results denoting lower environmental costs. 

Table 5 summarizes the results of the cost assessment for each pre-treatment used. The time costs of the UV pre-treatments varied according to the plastic type, with Medium for PS- and PET- and High for PE-derived plastics. The e-beam pre-treatments were all the least time-consuming (<24 h). Regarding the thermochemical pre-treatments, the time-cost assessment was high in all the scenarios, but additional experiments should be conducted to analyse lower time exposures due to the strong responses of the treatments, with >5 days probably required for optimal results. Similar behaviour was observed for the energy cost. The energy consumption of the UV treatments was lower for the PS and PET than for the polyolefins (LDPE and LLDPE). The e-beam pre-treatments were all the least energy-consuming (<15 KW/m^2^). However, the chemical treatment produced a medium energy consumption for all the polymers, except for the PS. The environmental cost was the highest for the thermochemical pre-treatments, including potential impact on GHG, water consumption, waste production and energy consumption, with the UV and e-beam having potential effects on the energy consumption. 

Based on these results, thermochemical treatments should be discarded due to their high time and high environmental effects for all the polymers. The opposite occurs using the e-beam treatment, with which the time, energy and environmental effects are low for all the polymers. Therefore, this new approach is recommended. As an alternative to e-beam, photo-oxidation could work very well for PS and PET, for which low energy and medium times are needed. However, for PE, longer times and higher energy are needed to enhance degradation. Therefore, it is not recommended for this type of polymer.

## 4. Conclusions

The results obtained show that the effect of the UV radiation was plastic-dependent, with more effective results obtained with UV-C for PS, LDPE and LLDPE and with UV-B for PET. The suggested duration of UV exposition to promote significant changes in plastics was also time-dependent, in the ranges of 30–90 h for PS and PET and 500–750 h for PE (LDPE and LLDPE), due to its more refractive structure. Considering all the pre-treatments studied, the thermochemical assay seemed to be the strongest approach, since it was able to affect not only the surfaces of the plastic probes, but all the plastic material. The ammonium persulfate/chromic mixture reagents produced stronger effects than the aqua regia, with time and temperature cooperative factors for disintegration/degradation. The e-beam radiation did not show consistent increases on the FTIR indexes, indicating less significant surface oxidation than that obtained with the UV. However, considering the TGA assessment, loss of integrity was promoted by the e-beam, given the decrease in the temperature to required achieve T98 and T95% conditions. Therefore e-beam pre-treatments must be considered, especially for PS and PE plastics; PET is the least affected with these integrity parameters. Thus, the overall efficiency of the plastic degradation obtained with the pre-treatments should be studied using a multicriteria approach, since FTIR assessments, in some cases, only consider oxidation processes on the plastic surface and do not show the potential integrity changes on the plastic probes. Therefore, considering the efficiency and cost–benefit offered by all the pre-treatments, thermochemical treatments should be discarded due to their long duration and high environmental impact. The e-beam and photo-oxidation treatments are more strongly recommended, since the time and energy requirements and environmental impact are low for the degradation of all the polymers studied. 

## Figures and Tables

**Table 1:** Details of the UV (types B and C) treatments.

Type UV	W × Chamber	Irradiated Surface (m^2^)	Power Consumption (kW/m^2^ h)
B	20	0.2479	0.161
C	16.7	0.2479	0.135

**Table 2:** Description of the thermochemical conditions (chemical reagents/temperature/time) to enhance plastic degradation.

Treatments	Code	Plastic Type	Conditions
(NH_4_)_2_S_2_O_8_ + H_2_SO_4_	AM	LDPE, LLDPE, LLDPE + LDPE, PP, PET, PS	6 and 12 days, 35 and 70 °C, concentrated and diluted (100% and 33%) reagents
K_2_Cr_2_O_7_ + H_2_SO_4_	CM	LDPE, LLDPE, LLDPE + LDPE, PP, PET, PS	6 and 12 days, 35 and 70 °C, concentrated and diluted (100% and 33%) reagents
HCl:HNO_3_:H_2_O 3:1:1, Aqua regia	AR	LDPE, LLDPE, LLDPE + LDPE, PP, PET, PS	6 and 12 days, 35 and 70 °C, concentrated and diluted (100% and 33%) reagents

**Table 3:** Values of FTIR carbonyl index used in the representative polymers after applying the e-beam pre-treatments.

E-Beam Pre-Treatment	Carbonyl Index			
	PS	LDPE	LLDPE	PET
0 kGy	0.309	0.039	0.070	0.999
100 kGy	0.277	0.063	0.087	1.002
200 kGy	0.331	0.065	0.094	1.002
500 kGy	0.373	0.097	0.125	1.004

**Table 4:** Summary of time–energy–environmental criteria established for pre-treatment cost assessment.

**Time consuming (T) Costs**	**Code**	**Assessment**
Low	T-L	<24 h
Medium	T-M	1–5 days
High	T-H	>5 days
**Energy consuming (E) Costs**	**Code**	**Assessment**
Low	E-L	<15 kW/m^2^
Medium	E-M	15–60 kW/m^2^
High	E-H	>60 kW/m^2^
**Environmental Cost (Env)**	**Code**	**Assessment**
Low	Env-L	0–1
Medium	Env-M	2–3
High	Env-H	3–4

**Table 5:** Results of cost assessment for optimal pre-treatments and plastic types.

Plastic Type	UV-B	UV-C	E-Beam	Thermochemical
PS	60 h 2.5 d 9.7 kW	45 h 1.9 d 6.1 kW	500 kGy <1 d 3.6 kW	AM ≈ CM > AR Concentrated > diluted 12 d > 6 d 35 °C ≥ 70 °C 8 kW
Cost-effectiveness assessment	T-M/E-L/Env-L	T-M/E-L/Env-L	T-L/E-L/Env-L	T-H/E-L/Env-H
PET	90 h 3.7 d 14.5 kW	30 h 1.3 d 4.0 kW	100–200 kGy <1 d 2.8 kW	AM ≈ CM > AR Concentrated > diluted 12 d > 6 d 70 °C ≥ 35 °C 16 kW
Cost-effectiveness assessment	T-M/E-L/Env-L	T-M/E-L/Env-L	T-L/E-L/Env-L	T-H/E-M/Env-H
LDPE	500–750 h 20–31 d 80–120 kW	750 h 31 d 101 kW	1000 kGy <1 d 4.9 kW	AR > CM > AM Concentrated > diluted 12 d > 6 d 70 °C ≥ 35 °C 16 kW
Cost-effectiveness assessment	T-H/E-H/Env-L	T-H/E-H/Env-L	T-L/E-L/Env-L	T-H/E-M/Env-H
LLDPE	500–750 h 20–31 d 80–120 kW	750 h 31 d 101 kW	1000 kGy <1 d 4.4 kW	CM ≈ AR > AM Concentrated > diluted 12 d > 6 d 70 °C ≥ 35 °C 16 kW
Cost-effectiveness assessment	T-H/E-H/Env-L	T-H/E-H/Env-L	T-L/E-L/Env-L	T-H/E-M/Env-H

AM: ammonium persulfate; AR: aqua regia; CM: chromic mixture.

## Data Availability

Data are contained within the article and supplementary materials.

## Supplementary Materials

The following supporting information can be downloaded at: https://www.mdpi.com/article/10.3390/polym16030359/s1, Table S1. Temperature of degradation and weight losses obtained with TGA analysis of the PS sample after photo-oxidation pre-treatments. Figure S1: Pareto charts for different FTIR indexes in the pre-treatment of PS with aqua regia. Table S2. Temperature of degradation and weight losses obtained with TGA analysis of the LDPE sample after photo-oxidation pre-treatments. Figure S2: Pareto charts for the different FTIR indexes in the pre-treatment of LDPE with ammonium persulfate. Figure S3: Pareto charts for other different FTIR indexes in the pre-treatment of LDPE with aqua regia. Table S3. Temperature of degradation and weight losses obtained with TGA analysis of the LLDPE sample after photo-oxidation pre-treatments. Figure S4: Pareto charts for other different FTIR indexes in the pre-treatment of LLDPE with ammonium persulfate. Figure S5: Pareto charts for the different FTIR indexes in the pre-treatment of LLDPE with aqua regia. Table S4. Temperature of degradation and weight losses obtained with TGA analysis of the PET sample after photo-oxidation pre-treatments.
