# Supplementary material for: Effect of Abiotic Treatments on Agricultural Plastic Waste: Efficiency of the Degradation Processes
Source: Polymers (Basel). 2024 Jan 29;16(3):359. doi: 10.3390/polym16030359 (PMC10857199; doi:10.3390/polym16030359)
Supplement: Supplementary file 1 [file polymers-16-00359-s001.zip › polymers-2814962-supplementary.pdf]

Supplementary Material

Table S1. Temperature of degradation and weight losses obtained with TGA analysis of the PS sample after photo-oxidation pre-treatments.

Sample name	PT[1]	Conditions	T at 98% wt (°C)	T at 95% wt (°C)	T at 50% wt (°C)	T_{max} (°C)	Residue (wt %)
PS_virgin	none	Non-treated	323.2	393.8	434.8	435.7	0.67
PS_UVB_60h	UV	UVB 60h	257.5	346.8	434.3	435.9	2.92
PS_UVB_90h	UV	UVB 90h	257.8	349.3	433.2	434.8	2.80
PS_UVB_120h	UV	UVB 120h	237.6	323.0	432.4	433.9	3.20
PS_UVC_30h	UV	UVC 30h	296.9	379.4	436.0	437.0	1.59
PS_UVC_45h	UV	UVC 60h	320.0	383.4	435.3	436.3	1.41
PS_UVC_60h	UV	UV C90h	307.0	379.8	435.4	437.1	1.43
PS_e-beam_600	e-beam	600 Gky	303.4	384.5	430.6	431.3	2.17

[1] type of pre-treatment.

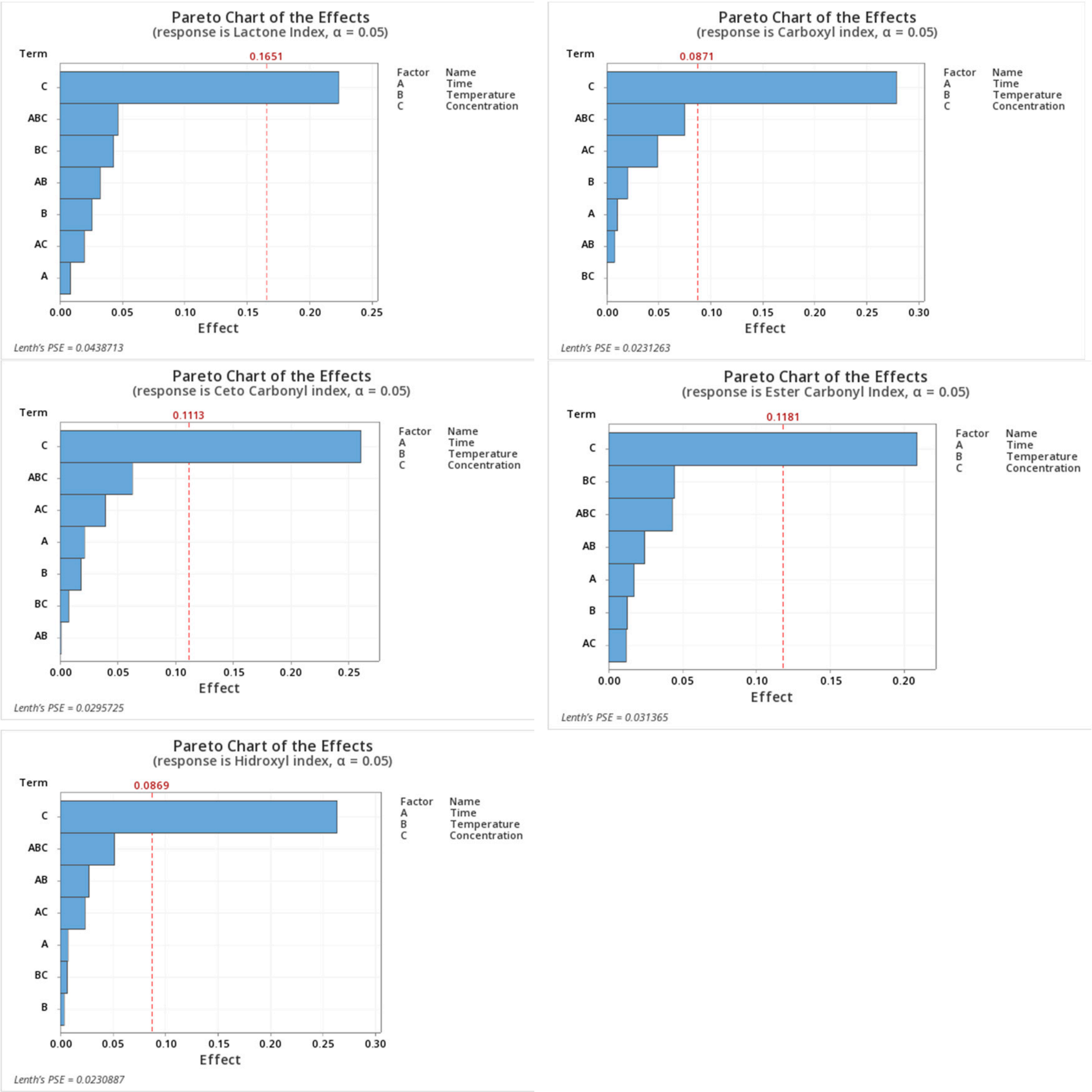

Figure S1. Pareto charts for different FTIR indexes in the pre-treatment of PS with aqua regia.

Table S2. Temperature of degradation and weight losses obtained with TGA analysis of the LDPE sample after photo-oxidation pre-treatments.

Sample name	PT[1]	Conditions	T at 98% wt (°C)	T at 95% wt (°C)	T at 50% wt (°C)	T_{max} (°C)	Residue (wt %)
LDPE_virgin	none		412.7	427.7	466.0	470.2	4.94
LDPE_UVB_250h	UV	UVB 250h	417.0	428.2	465.4	470.3	5.17
LDPE_UVB_500h	UV	UVB 500h	406.7	422.6	462.6	467.5	2.79
LDPE_UVC_250h	UV	UVB 250h	402.8	422.2	466.6	472.1	3.63
LDPE_UVC_500h	UV	UVC500h	378.5	416.1	464.2	469.1	3.90
LDPE_e-beam_300(1)	e-beam	300(1)	393.7	414.3	462.5	468.8	1.75
LDPE_e-beam_400(1)	e-beam	400(1)	394.9	414.1	462.9	469.4	1.97
LDPE_e-beam_400(2)	e-beam	400(2)	392.6	415.1	465.1	472.0	1.03
LDPE_e-beam_450(2)	e-beam	450(2)	391.2	414.6	465.3	471.8	1.02

[1] type of pre-treatment.

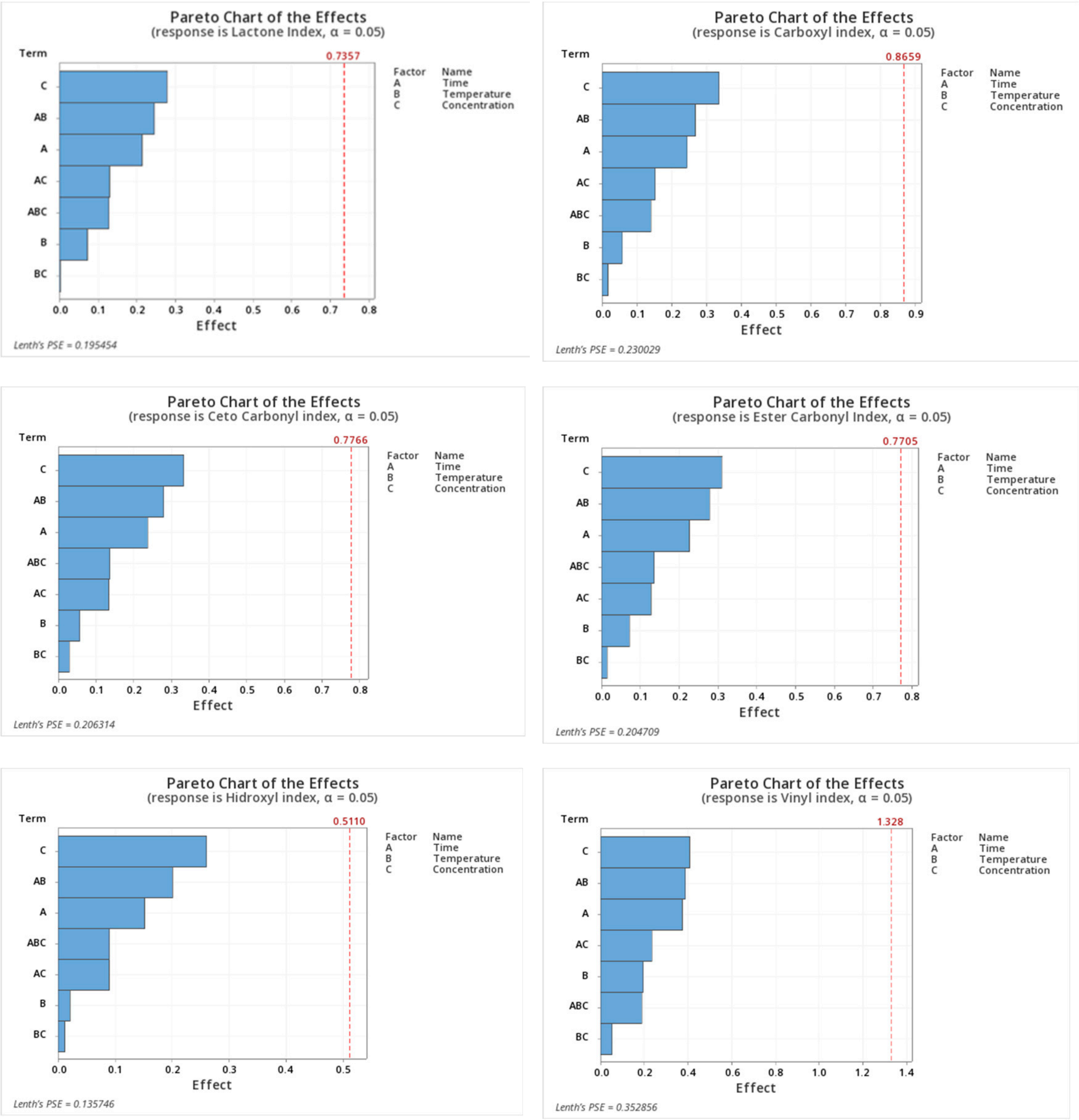

Figure S21. Pareto charts for the different FTIR indexes in the pre-treatment of LDPE with ammonium persulfate.

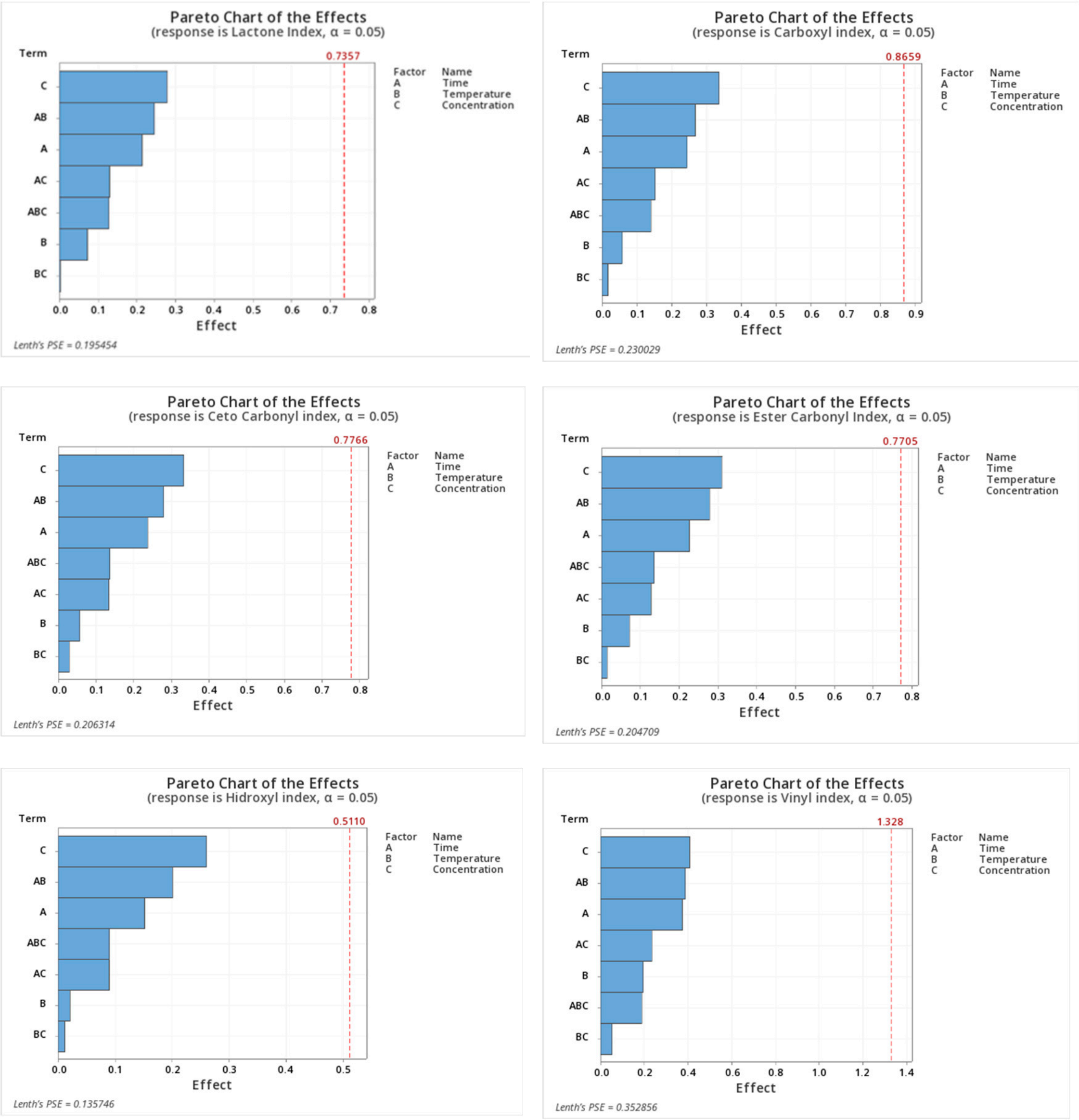

Figure S3. Pareto charts for other different FTIR indexes in the pre-treatment of LDPE with aqua regia.

Table S3. Temperature of degradation and weight losses obtained with TGA analysis of the LLDPE sample after photo-oxidation pre-treatments.

**Sample name**	**PT[1]**	**Conditions**	**T at 98% wt (°C)**	**T at 95% wt (°C)**	**T at 50% wt (°C)**	**T_{max} (°C)**	**Residue (wt %)**
LLDPE_virgin	none		417.4	431.7	469.9	474.2	3.10
LLDPE _UVB_250h	UV	UVB 250h	417.3	433.3	469.7	473.9	2.75
LLDPE _UVB_500h	UV	UVB 500h	412.8	428.3	467.1	471.2	4.19
LLDPE _UVC_250h	UV	UVB 250h	413.5	427.7	467.9	472.6	3.77
LLDPE _UVC_500h	UV	UVC500h	365.7	412.2	464.9	470.7	2.81
LLDPE_e-beam_300(1)	e-beam	300(1)	406.9	425.1	468.0	473.9	1.17
LLDPE_e-beam_400(1)	e-beam	400(1)	407.1	424.7	467.1	473.0	1.21
LLDPE_e-beam_250(2)	e-beam	250(2)	405.8	423.9	467.3	473.1	1.35
LLDPE_e-beam_250(2)	e-beam	250(2)	407.8	424.8	467.2	473.1	1.40
LLDPE_e-beam_300(2)	e-beam	300(2)	403.5	419.7	464.9	471.0	2.88
LLDPE_e-beam_350(2)	e-beam	350(2)	405.3	423.3	466.5	472.3	1.20
LLDPE_e-beam_400(2)	e-beam	400(2)	407.4	424.1	465.5	470.7	1.31
LLDPE_e-beam_450(2)	e-beam	450(2)	402.6	421.2	464.0	469.5	1.52
LLDPE_e-beam_333(3)	e-beam	300(2)	417.4	431.7	469.9	474.2	3.10

[1] type of pre-treatment.

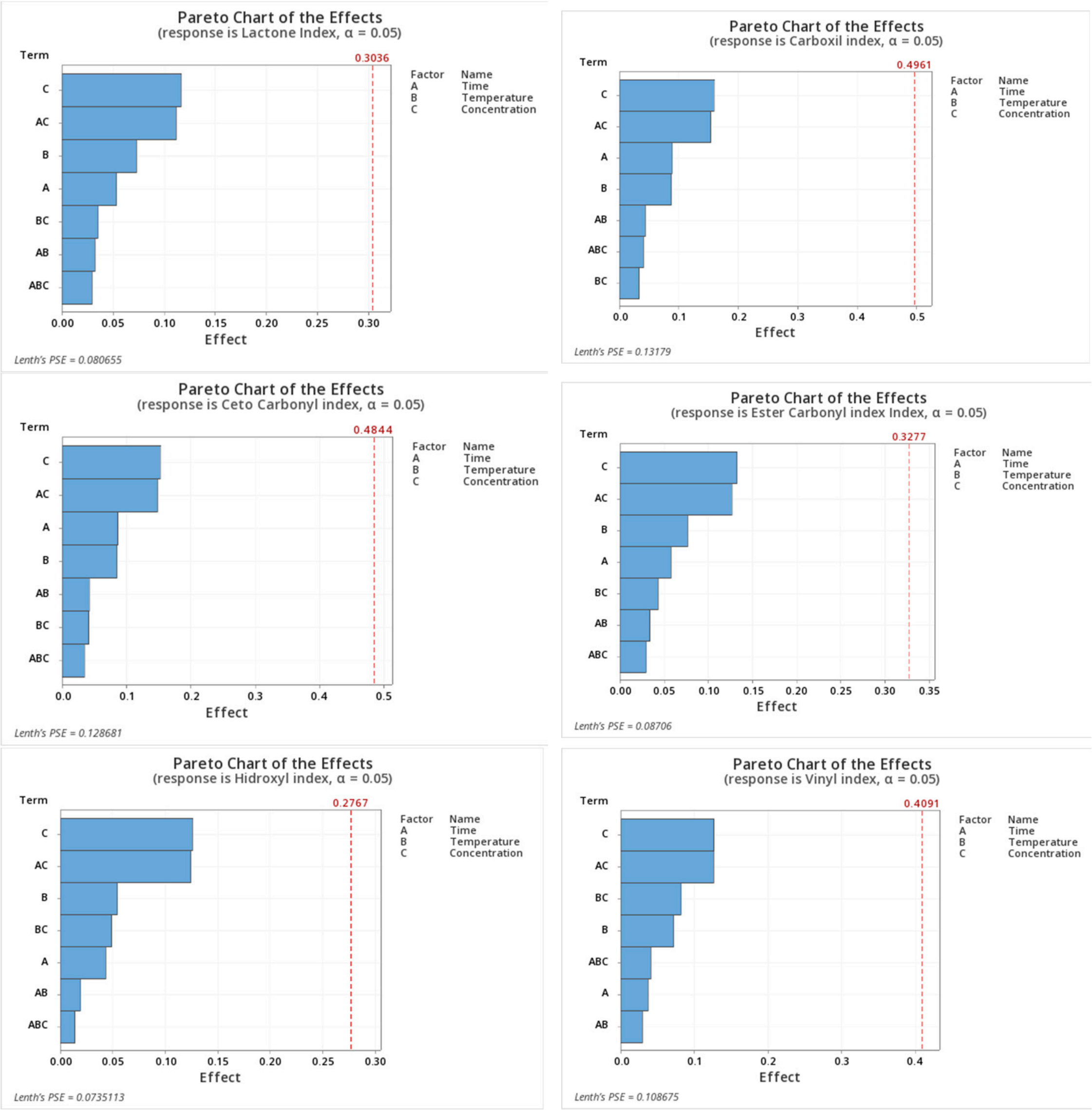

Figure 2S4. Pareto charts for other different FTIR indexes in the pre-treatment of LLDPE with ammonium persulfate.

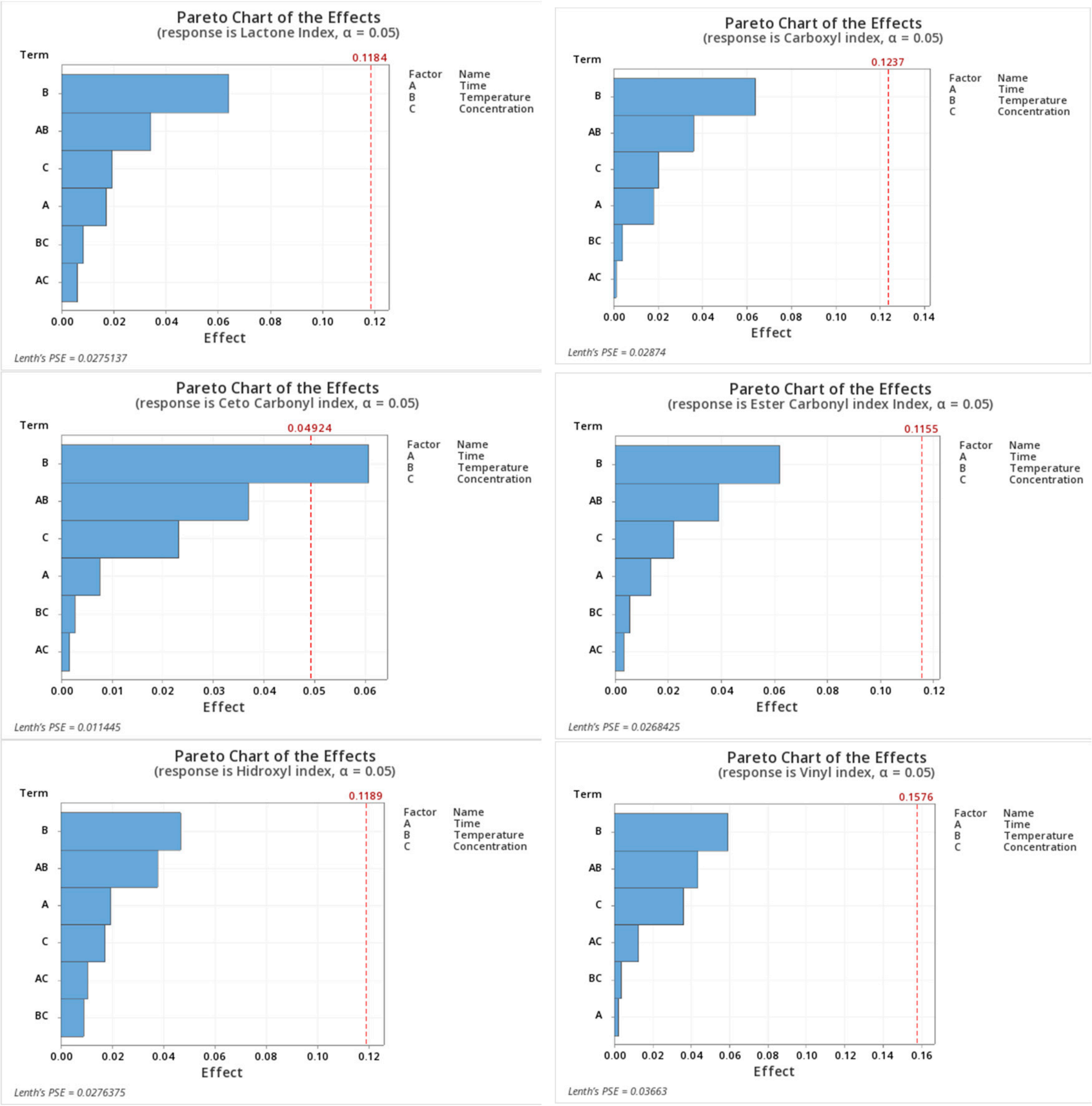

Figure 3S5. Pareto charts for the different FTIR indexes in the pre-treatment of LLDPE with aqua regia.

Table S4. Temperature of degradation and weight losses obtained with TGA analysis of the PET sample after photo-oxidation pre-treatments.

Sample name	PT[1]	Conditions	T at 98% wt (°C)	T at 95% wt (°C)	T at 50% wt (°C)	T_{max} (°C)	Residue (wt %)
PET not treated	none	Non-treated	381.7	394.3	432.2	432.5	11.66
PET _UVB 60h	UV	UVB 60h	380.0	394.2	432.9	433.4	11.73
PET _UVB 90h	UV	UVB 90h	376.4	392.9	431.6	432.0	11.85
PET _UVB 120h	UV	UVB 120h	377.6	393.2	431.5	432.0	12.06
PET_UVC 30h	UV	UVC30h	382.3	394.6	432.2	432.6	11.71
PET_UVC 45h	UV	UVC60h	383.3	394.7	431.9	432.5	11.71
PET_UVC 60h	UV	UVC90h	383.6	395.7	432.0	431.9	11.82
PET _e-beam_300(2)	e-beam	300(2)	377.2	394.2	434.6	435.5	12.47

[1] type of pre-treatment.
